# Investigating User Identification in Remote Patient Monitoring Devices

**DOI:** 10.3390/bioengineering4030076

**Published:** 2017-09-13

**Authors:** Brian Ondiege, Malcolm Clarke

**Affiliations:** Department of Computer Science, College of Engineering, Design and Physical Sciences, Brunel University London, London UB8 3PH, UK; Malcolm.clarke@brunel.ac.uk

**Keywords:** elderly patient technology, ehealth, elderly patient behaviour, Patient Technology Acceptance Models

## Abstract

With the increase in the number of people having a chronic disease, there is an increase in households having more than a single person suffering from the same chronic illness. One problem of monitoring such patients in their own home is that current devices have a limitation in the number of people who can use a single device. This study investigates the use of Near Field Communication (NFC) for identification in a multi-user environment. Methods: A mixed-method qualitative and quantitative approach was adopted, including focus groups, observations and a field trial. Data were collected in three phases. In Phase 1, five focus groups were conducted with patients to determine their beliefs, concerns and issues with using identification in remote patient monitoring devices. In Phase 2, participants were given a blood pressure monitor modified to include an NFC reader to enable identification. The modified device was given to patients living as a couple in the same household and both suffering from hypertension. Both patients used the device for a period of two weeks to observe their acceptance of the technology and determine their experience of usage. A total of 40 (20 couples) patients participated in the trial. Non-adherence to the full monitoring regimen was low and was mainly due to usability issues or commitments taking them away from the home and thus unable to take readings. After the trial period participants were invited to discuss their experiences with the technology in a focus group discussion (Phase 3), a total of five focus groups were conducted. Focus group discussions with the patients revealed that most participants liked using the system and were not apprehensive towards Healthcare Information Technology (HIT). The participants also had suggestions for improvements that could be made to the modified blood pressure monitor (such as, rechargeable in place batteries, integrate the components, easier to use cuff, and increased sensitivity of the NFC reader) that might improve the overall experience of the proposed technology and its acceptance. Conclusion: The study proposes a new framework, the Senior Patient Technology Acceptance Model (SPTAM) that offers an understanding of the needs of the elderly towards technology use and the factors that influence its acceptance. SPTAM emphasises that involving the patient in the early stages of development can lead to a more user-centred technology and help in identifying any underlying issues at an early stage, thus avoiding adding features which patients do not need. The findings from this empirical research can be used as recommendations to improve current RPM devices, save the NHS costs, inform standardization groups.

## 1. Introduction

The number of people aged 60 or over is expected to pass the 20 million mark by 2030. The number of people aged 65+ is projected to rise by nearly 50% (48.7%) in the next 17 years to over 16 million [1]. These demographics present challenges to the healthcare sector, with the ageing population being the highest consumers of healthcare. This increase has seen households having more than one person suffering from the same chronic illness [2,3]. These present challenges in home monitoring as current devices have a limitation to the number of people who can share a single device.

Literature shows that the elderly people are apprehensive towards the use of Healthcare Information Technology (HIT) [4,5,6,7]. This can be a hindrance to technology acceptance and adoption. A recent study conducted with healthcare professionals on their perception of the security of Patient Health Devices (PHD) highlights the need to address patient identification [8]. To address these issues, an appropriate identification technique built into RPM devices can be beneficial in settings such as care homes for the elderly and households that have two or more people suffering from the same chronic disease. This will support a large number of users being monitored, potentially with reduced costs associated versus having multiple devices and systems; however, with the current architecture, this possibility is limited [9]. This creates urgency for an identification technique that is designed towards the needs of the frail elderly. This will involve investigating factors that influence acceptance of technology among the frail elderly to ensure any technology that is presented to them is accepted readily.

### 1.1. Terminologies 

Remote Patient monitoring devices—technology that allows patients to use medical devices outside of conventional clinical settings, i.e., their homes, which may increase access to care and decrease healthcare delivery costs [10].

Telehealth—“is the remote exchange of data between a patient at home and their clinician(s) to assist in diagnosis and monitoring typically used to support patients with Long Term Conditions, uses technology to provide services that assist in the management of long term health conditions, including, hypertension, Chronic Obstructive Pulmonary Disease (COPD), Chronic Heart Failure (CHF), Diabetes and Epilepsy” [11].

### 1.2. Theoretical Framework for Technology Acceptance

This section looks at theoretical frameworks.

#### 1.2.1. Technology Acceptance Model

**Figure 1:**
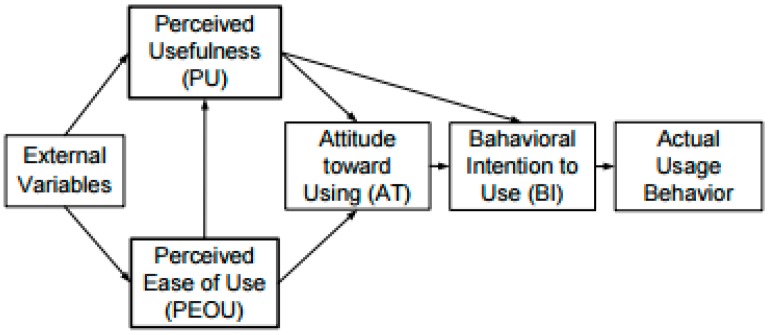
Technology Acceptance Model.

Technology acceptance refers to favourable reception and continuous use of newly introduced technologies, devices and systems [12] (Figure 1).

The level of acceptance, according to Renaud et al. [12], is an attitude towards a particular behaviour and relates to the feelings of the person (negative or positive) about the behaviour and the extent to which this behaviour is affected. TAM incorporates six factors, as shown in Figure 1.

Perceived usefulness (PU) refers to the extent to which a user believes that a certain system can improve their job performance.

Perceived ease of use (PEOU) refers to the extent to which a user accepts that using a given technology would be easier to use.

External variables (EV), such as demographic variables, influence perceived usefulness (PU) and perceived ease of use (PEOU).

Attitudes towards use (A) is defined as the user’s desirability to use the system.

Perceived usefulness (PU) and perceived ease of use (PEOU) are the sole determinants of attitude towards the technology system.

Behavioural intention (BI) is predicted by attitude towards use (A) combined with perceived usefulness (PU).

Actual use (AU) is predicted by behavioural intention (BI).

TAM is somewhat limited, since the only determining factor leading to actual system use is depicted as behavioural intention to use, which is unrealistic in our context.

#### 1.2.2. Theory of Reasoned Action

Theory of Reasoned Action (TRA) is a comprehensive theory that focuses on a number of psychological variables that can influence behaviour such as attitude, intention, subjective norm, normative, behavioural and control beliefs, and perceived behavioural control. The TRA postulates that a user’s intention or motivation determines their behaviour, whereas the intention is influenced by the user’s attitude formed through negative and positive evaluations established from behavioural beliefs and subjective norms [13]. Therefore, Ajzen [13] explained that the main assumption of TRA is that users are likely to engage in a behaviour when they have a high intention that is raised when they evaluate a behaviour positively, that is, attitude, and believe that others want them to engage in it, that is, subjective norm. Ogden [14] argues that TRA focuses mainly on analytic truth, in which the conclusions are not supported by observation.

These theoretical models are limited in their scope and area of application and do not consider cognitive impairment, cost, age, social influence and other factors that influence acceptance and adoption of technology for the elderly [12]. These make these frameworks unsuitable for studying and focusing on technology adoption and acceptance among the elderly for RPM purposes.

The aim of this study is to investigate and address current limitations and experience of usage of two or more people sharing a single RPM device. 

The aim of this research is realised through the following objectives:

- Develop RPM device modified with identification technology.
- Involve elderly in the design and development process.
- Investigate the experience of elderly people with the modified RPM to determine issues of continued use and if they would continue to use.

This study draws concepts and techniques from two research projects funded by the European Union (EU), Integrated Network for Completely Assisted Senior Citizen’s Autonomy (INCASA) and Remote Accessibility to Diabetes Management and Therapy in Operational Healthcare Networks (REACTION). The INCASA project involved developing systems that support the aging population and facilitate them to stay longer and healthier in their own homes, with the combined monitoring of health and activities of daily living (ADL) through unobtrusive monitoring [15]. The REACTION project involved development of an intelligent platform to provide remote monitoring to support long-term management of people with diabetes [16].

## 2. Materials and Methods 

This study utilised mixed method methodology: qualitative and quantitative. The rationale for using mixed method approach in the present research study was to explore and describe the opinion of patients suffering from chronic disease, their behavioural habits, and their perceptions of using NFC for identification in RPM devices. A qualitative approach was appropriate to capture the patients’ beliefs, needs, and attitudes towards the use of NFC in RPM devices, which in turn may determine if they will be willing to use the technology. A quantitative approach was appropriate in capturing failed observations during the trial period.

The rationale for using focus groups is because they are most suitable to use when the objective is to understand better how people consider an experience, idea or event because the discussion in the focus group meeting can be made effective in providing information about peoples’ perceptions, how they feel, or the ways in which they behave.

### 2.1. Study Design

This study was conducted in three phases: Phase 1 investigated different identification techniques with patients through a focus group discussion to determine their perceptions and preferences about the identification technologies. In Phase 2, Patients participated in a field trial by taking the modified prototype device for a period of two weeks. Observed data from the trial were analysed to identify frequency of usage and failed observations. Issues observed in this phase can be used to improve future RPM devices. In Phase 3, the post-trial focus group, participants who participated in the field trial were invited after the field trial period to share their experiences with the technology to determine if they would continue to use and how it can be improved to increase acceptance. 

#### 2.1.1. Phase 1: Pre-Trial Focus Group

Three locations were chosen for the study: Chorleywood Health Centre, a2Dominion and ISLAD. ISLAD in Slough and a2Dominion in Northwood are both residential homes for the elderly. Five focus groups were conducted. Chorleywood Health Centre is an average sized general practice in Chorleywood. Chorleywood Health Centre was chosen due to its experience in participating in research for frail elderly technology, and existing close collaboration with Brunel University. Phase 1 of the study was conducted for a period of one month between September 2016 and October 2016.

##### Sample

Participants were selected from the population of frail elderly living with chronic disease, such as diabetes or hypertension, and who were considered able to use the home monitoring devices.

The inclusion criteria for participation in Phase 1 of the study were:

- Able to communicate in English language
- Age 64 years or above
- Registered with Chorleywood Health Centre or living in either of the two residential homes, a2Dominion and ISLAD
- Currently suffering from diabetes or hypertension.

Elderly participants were chosen because they are the greatest users of RPM devices and many suffer from chronic disease. One hundred people were identified as eligible to participate in the study, but only 58 took part. Five focus groups were conducted. 

#### 2.1.2. Phase 2

Phase 2 involved patients testing the prototype equipment in the home environment for a period of two weeks. The 14-day period has been shown to capture well the long-term value of blood pressure monitoring in hypertensive patients [17]. Patients for this phase were selected only from Chorleywood Health Centre as the phase included the collection of clinical data. Moreover, the current guidelines from the National Institute for Care Excellence (NICE) and the national framework for management of diabetes (which require clinical measurement of blood glucose and blood pressure [18]), requires patients to take one blood pressure measurement each day for 14 days using the home monitoring devices [19]. This study was designed to work following current clinical work flow and guidelines such as NICE. A database for patients in Chorleywood Health Centre was used to search for patients who were living as a couple within the same household and both were suffering from the same chronic disease. Sixty participants were identified as suitable for the study, of which 40 participants (20 couples) participated in the trial period. This study used purposive sampling. This involved the researcher selecting those participants who would be included in the study. The rationale for this approach was that the researcher sought knowledge about patients’ behavioural habits associated with use of NFC for identification in RPM devices to determine their opinion, which the participants would provide by virtue of having a lived experience with the technology.

Each patient was provided with a health kit that comprised the blood pressure meter, an NFC scanner, an NFC key fob or NFC card, and a (laptop) PC. Data from the BP were transferred wirelessly to the PC, and used a ZigBee Universal Serial Bus (USB) dongle to pass the data to the PC. The NFC scanner was connected to the PC by USB.

Software was developed for the PC to capture the NFC ID (User identification) from the NFC scanner and the blood pressure (BP) data coming from the BP monitor. The readings associated with the user identification given at the time of the reading were stored together on the PC for later upload to a database for analysis.

#### 2.1.3. Phase 3: Post Trial

After the trial period, the couples were invited to a focus group meeting to discuss their lived experience while using the modified RPM device. Five focus groups were conducted in the post-trial phase.

Protocol for identification and blood pressure monitoring trial.

Each day:

- The patient touches their card/tag on the NFC reader until the LED indicates the card/tag has been recognised—the identification is sent to the PC.
- The patient takes their blood pressure using the modified prototype monitor—the measurement is sent to the PC.
- The PC saves the measurement and identification in a local database.

Data are extracted from the PC at the end of the trial period for later analysis. Any deviation that the patient has made from the protocol will be seen at this stage. It was explained to the participants that their data were not being monitored by the health centre on a daily basis, and the data were not going to be used for clinical decision.

### 2.2. Ethics and Consent

The study received ethical approval from Brunel University Ethics Committee. The objective of the research was explained to each participant, and informed consent was obtained prior to starting the interview.

### 2.3. Data Collection Instrument and Method

In the present study, little knowledge existed on the research phenomenon to formulate a hypothesis, thus inductive and deductive approaches were used. Coding of the data was performed using QDA miner, a qualitative data analysis tool. Inductive and deductive coding techniques were used to identify the topics relating to technology acceptance among the elderly; the major expressions relating to identification techniques, their experiences and perception of identification techniques, and the dynamics that are brought about by ageing and disease. 

The major expressions were then formulated into meanings and categorized into themes and sub-themes. The resulting themes and sub-themes were then combined into an all-inclusive and in-depth description of the phenomenon under investigation.

All focus group interviews were digitally recorded and later transcribed. Thematic analysis was used on the data to identify the important themes and to understand the significance of the themes identified in advance from the literature survey.

## 3. Results

All focus group discussions were transcribed and analysed thematically using the thematic data analysis technique. The focus group data were coded inductively and the codes were categorised to form larger themes. Themes were selected according to the frequency and the number of times mentioned by the patients in the data set within the transcripts.

### 3.1. Technology Assessment

Phase 1 involved assessment and evaluation of identification technologies. Participants discussed their experiences, perception, and preference of identification technologies. None of the participants selected PINs as their preferred choice of identification, as participants were concerned about the ability to remember them. 

For fingerprints, some participants saw biometrics to be the most secure of the identification techniques but were concerned about the cost of implementation and ensuring reliable scanning and identification. In addition, some participants saw fingerprints as a monitoring mechanism by the government. 

Three types of NFC identification technologies were discussed: wristband, key fob and card. None of the participants selected the wristband as their ideal identification technique. Some participants were apprehensive towards wristbands as it reminded them of being in hospital, while others saw the wristband being childish or something a teenager would wear. The majority of the participants who selected the key fob stated that this was due to the ability to fit into their key chain, whereas those that did not select the key fob stated that they did not want to add additional bulk to their set of keys. The majority of the patients who selected the card stated that this was due to the ability of the card to fit into their wallets and its durability compared to magnetic strip cards, whereas those that did not select the NFC card were concerned about mixing up their cards with their partner’s card at home. Cost was a dominant concern as a determinant for technology acceptance among many of the patients. Based on the analysis of the results from the focus group, this study selected NFC as the identification technique, with the selection of the NFC card or key fob as the identification tag.

### 3.2. Themes Phase 1: Pre-Trial

#### 3.2.1. Patient Characteristics

The patient characteristics theme was comprised of six sub-themes: disease and physiological characteristics, attitudes to learning and technology self-efficacy, exploration, patient involvement, education, and training and reviews. These sub-themes are reported below.

##### Sub-Theme: Disease and Physiological Characteristics 

The findings of the focus group revealed that challenges associated with physiological characteristics and activities, such as cognitive capabilities, hearing, sight, and mobility resulting from the ageing process, were imperative to participants. For example, some of the participants expressed concerns regarding their ability to calculate properly the average of their blood glucose readings (as required before seeing the diabetes nurse every three months), whilst other participants had to seek help from their spouses. Participants also articulated concerns with using certain devices, such as handling the measuring sticks of the glucose meter with arthritic fingers. These issues were raised by participants who explained they believed this was due to ageing and physiological processes. A participant expressing these issues said:

“I don’t know how to calculate my readings well as I am getting older now and my numerical skills are all gone and I have arthritis. My wife helps in calculating my readings and recording them in a small book which I use when I go to see the diabetic team.” (FGD1).

The findings also showed that, as people grow older, there is a decline in working memory, which can make it difficult for the elderly to undertake certain tasks. When asked, what negatives were associated with the use of a PIN (Personal Identification Number), some of the participants expressed concerns with their memory capacity and ability to remember using the PIN and passwords. A participant expressed this issue as:

“I am always forgetting what happens when I forget my PIN number. Will that mean I won’t be able to take a reading on that day?” (FGD4).

Another participant who raised the same issue said:

“I am always frustrated with PINs and passwords. I get them confused, and then try to change them, which has become an endless cycle for me.” (FGD5).

##### Sub-Theme: Attitude to Learning and Technology Self-Efficacy

The findings showed that factors affecting attitude were very important in determining technology acceptance. Most of the participants acknowledged that the ability of the technology to help them to manage their condition was the most important factor that would influence them in using the proposed technology. Expressing this desire, a participant said:

“Anything that can be done to help improve my condition (I) am open and willing to learn. We have seen amazing things that technology can be able to do.” (FGD3).

“If this technology can help us manage our conditions or even possibly help us get better, then I would go for it.” (FGD3).

The findings also revealed that the complexity of technology could deter some patients from using it, which was evident from some participants who wished to have a system that makes life easier for them. This was expressed as follows:

“Technology should be simple and as easy to understand as possible. Adding a lot of complex features makes life harder for us, as we are struggling with other things as well and our ability to learn is not what it used to be.” (FGD2).

##### Sub-Theme: Exploration 

The findings showed that the first impression of a user during first contact with technology results in judgement on the ease of use of the technology. This was evident from the participants who were introduced to a technology with which they were familiar. Having lived in London, the majority of the participants were familiar with the use of either contactless payment, or had used the local transport system, which uses the Oyster Card that is based on NFC technology. In response to the experiment on the device and when asked to give their feedback on its ease of use, some participants said:

“It’s similar to an Oyster experience, it’s quicker. Majority of the elderly are using this technology on a daily basis for travelling.” (FGD3).

“I don’t think I would have a problem with it but the connections are many.” (FGD1).

“It’s easy to use, gets you authenticated faster.” (FGD2).

While expressing their past experience using a wristband for identification and how it reminded them of being in a hospital, some participants stated:

“I wouldn’t want to wear a wristband in my house; it reminds me of the time I was in hospital.” (FGD2).

“I wouldn’t wear the wristband, it looks childish, something that a teenager would wear.” (FGD4).

##### Sub-Theme: Patient Involvement

The findings showed that the majority of the patients expressed their satisfaction at being involved in the process of development, which made it easier for them to relate when they started using the device. However, some participants proposed the need to collaborate with the device manufacturers so that they can understand the needs of the elderly, as most device manufacturers do not consider the needs of the elderly. Participants suggested that:

“The clinicians and technology developers should be doing this often by involving patients; sometimes technology developers add additional features which are not even necessary.” (FGD4).

However, some participants suggested that manufacturers should collaborate with the clinicians to understand the requirements of patients. A participant said:

“There should be a close collaboration between device manufacturers and clinicians to better understand our needs.” (FGD3).

##### Sub-Theme: Education, Training and Reviews

The findings revealed that some participants acknowledged the importance of education gained through various resources and by talking with different people as the best way to manage their condition, and which would help them to accept and cope with their disease. In response to the question regarding what would influence them to decide to use the proposed technology, the majority of participants saw education as an important factor. Participants expressed this as follows:

“When I was diagnosed with diabetes I was in a lot of pain and was really depressed for a long time. My doctor recommended me to go through a pain management course to help me in managing my pain and how to cope with the disease as I was going to have it for life. The course helped me as I don’t push myself much and I have accepted my current situation. And I only recently started taking my insulin and am reading books on dietary management as well. I would be open to use this technology if I understand it better.” (FGD3).

In addition, participants raised concerns on usability issues and the need for specialised training in using the proposed technology. A participant expressed the concerns by saying:

“Training on use of this technology and its benefits will be important as some people are not aware of this technology and its benefits. This technology can be used to address the challenges faced by the NHS.” (FGD4).

Another participant said:

“I had to prick my skin several times, my fingers were really sore, wouldn’t use that product again. Nowadays I ensure I am more updated on the product I use as I check for reviews from different sources.” (FGD5).

#### 3.2.2. Theme 2: Technology Concerns

The concerns about the technology theme included four sub-themes: trust and security, cost, reliability and performance, and benefits of the technology. These sub-themes are presented as follows.

##### Sub-Theme: Technology Trust and Security

When asked, what issues would be of concern if their physiological data were transmitted electronically using the modified RPM technology, most of the patients showed no real concern about security issues associated with sending their data electronically. Participants expressed this trust in the technology as:

“Am comfortable in sending my data electronically, don’t see any issues with it.” (FGD1).

“I don’t care who gets access to my records as long as they help in managing my condition or even help me get better, who knows.” (FGD2).

It was also found that most patients did not see the need for addressing security as they did not see the value of confidentiality while sending physiological measurements such as BP readings. Participants expressed this as:

“I don’t care about security issues; it’s only my readings that I will be sending.” (FGD5).

However, a few patients expressed the importance of security and confidentiality in the management of their healthcare records and expressed that only authorised people should have access to privileged information. A participant stressed that:

“Why should I show this information with Tom & Harry? I think security is very important; you don’t want your personal information to be disclosed to everyone.” (FGD1).

Another participant stated:

“Not worried about 100 percent security only authorised people.” (FGD3).

##### Sub-Theme: Cost

The findings showed that some participants expressed the importance of cost in acceptance of technology. With NHS services becoming strained and critical services being cut, most elderly patients showed awareness of the current crisis in healthcare. A participant stated:

“Anything that would be the easiest and cheapest for NHS will be sensible to use.” (FGD3).

Majority of the patients were positive regarding the ways in which the proposed technology was going to help in cutting costs for the healthcare service. 

One participant expressed this optimism by saying:

“This technology sounds like a very efficient and cost saving technology.” (FGD4).

Another participant added:

“RPM sounds like a very efficient and cost saving technology.” (FGD2).

The findings also showed that, although most participants acknowledged biometrics provided security to the technology, many had concerns regarding its cost. A participant said:

“I find biometrics to be secure but am thinking it will be very expensive to implement.” (FGD3).

##### Sub-Theme: Reliability and Performance

The findings showed that trust of the technology could be associated with the role it played in their health, and the reliability and accuracy of the device in use.

In addition, some participants expressed interest in the reliability of the system to work properly and they were concerned about sending their readings when there was a problem with their broadband. These concerns were raised as follows:

“What measures are there to ensure that when the broadband fails the readings are going to be sent? What happens to those readings that haven’t been sent?” (FGD4).

It was also found that participants assessed the ability of the device to do its job as a key determinant for acceptance and use of the technology. A participant stressed this issue and said:

“Is it going to do the job?” (FGD1).

In addition, some participants expressed the importance of simplicity for acceptance and use of technology, which they reported in response to questions asking what things were important to them when it comes to selecting a technology.

A participant responded:

“Ideally anything that is simple to use would be a great deal.” (FGD3).

##### Sub-Theme: Technology Benefit

The findings revealed that participants new to RPM were impressed with the ability of RPM to transmit their physiological data, which the clinician could view and determine when an intervention would be required.

A participant responded:

“Sounds so futuristic, but it should be linked up to clinician’s systems for it to be significant.” (FGD2).

Participants acknowledged the importance of patient identification. A participant suggested:

“You don’t want the doctors to prescribe something that is not right with you, there are dangers.”

However, some participants expressed the importance of the technology and support from their spouses as significant in participating in the study, together with the importance of the proposed technology to their situations. A participant expressed these issues as:

“Me and my partner suffer from hypertension, this will be really beneficial to us as we can be encouraging each other in taking our measurements and taking control of our conditions.” (FGD4).

Another participant said:

“Chronic disease can be dreadful; me and my partner are both suffering from diabetes. If this technology can allow both of us to be monitored and save the NHS costs, then we would go for it.” (FGD5).

It was also found that some of the participants who were new to RPM expressed frustration in the current diabetes recording system, which involved recording the glucose readings in a small book and making a visit every three months to see the diabetic team to discuss their condition. The issues were expressed as:

“Sometimes am not even bothered about recording my readings because I forget. If this technology will remind me, and the doctors can be checking up on me without going there, then that would be brilliant.” (FGD1).

The findings also showed that some of the participants selected the key fob because of its convenience to their lifestyle and ability to fit in their pockets. These advantages of the technology were reported as:

“Fits in the pocket, not too bulky.” (FGD2).

#### 3.2.3. Theme 3: Clinician Trust

The findings of the focus groups revealed that trust in clinicians was a very significant factor in determining acceptance of technology among the frail elderly. Participants had differing views about their relationship with their healthcare professionals and the way they influenced their decision on use of technology. The findings showed that some participants would only use the proposed technology if their diabetic team recommended it to them. As a participant said:

“If my diabetic doctor saw the need for me to use RPM, then I would because I know they will only recommend what will be beneficial to me.” (FGD1).

However, some patients raised concerns that showed their mistrust in healthcare professionals in relation to their request to see their own medical records. A participant expressing this issue said:

“It’s like sometimes they are hiding something from us. They only show us what we want to see, so sometimes I take an initiative before I see my doctor to search for more information on my signs and symptoms so that am more informed before my visit. Are you aware that 30% of deaths in hospitals are caused by doctors?” (FGD2).

### 3.3. Phase 2: Trial Data Analysis

In Phase 2 of the study, the participants were asked to use the equipment in the home environment for a period of two weeks. During this period, the behavioural habits on its use were observed. The observed data measurements collected Phase 2 were analysed quantitatively.

#### 3.3.1. Demographics

Table 1 provides the demographics of the participants of the study. The data include gender and the number of participants.

#### 3.3.2. Observed Data Analysis

This study used automatic logging to capture every time the user used NFC identification and took a BP reading. Usage of the equipment was automatically recorded to determine correct and incorrect usage, or if the equipment was not used that day.

#### 3.3.3. Overall Usage

Table 2 shows the usage errors made by the patients. The percentage for each day is based on the expectation of receiving 40 readings per day, one per patient.

#### 3.3.4. Analysis of Multiple Use of the Same Card

Days 1 and 14 had the same user who used the same tag more than once. Days 5 and 8 had the same user who used the same card more than once in two days, while the remaining users who made the same error made it on one occasion only during the trial period.

#### 3.3.5. Analysis of Tag Not Placed Properly on the Reader

One user made these error three days consecutively, Days 1, 2 and 3 as they failed to place their tags properly on the reader. On Days 2 and 8, another user failed to place their tags properly on the reader, while on Days 11, 13, 5 and 2, the error was only made once during the trial period.

#### 3.3.6. Errors Made

Two main forms of errors were observed in this study:

- Multiple use of the same card; and
- Card not placed properly on the reader (reading alone).

#### 3.3.7. Multiple Use of the Same Card

The participants were given strict instructions that they were to take only one reading per day, and therefore would use their card only once per day. Multiple use of the same card on the reader on the same day would indicate that a user has used a card that was not theirs. This might occur intentionally or unintentionally, as the following examples highlight.

##### Example

Two couples expressed frustrations about the sensitivity of the key fob and its unreliable operation. During analysis of the results, it was found that the same tag had been used twice on the same day but each use was accompanied by a widely different BP reading, indicating that one person had used their partner’s tag.

#### 3.3.8. Tag not Placed Properly on the Reader

This error occurs when the user fails to place their tag properly on the reader and results in their tag not being read by the reader. In the trial, there was one case of the card not being placed properly on the NFC reader was reported.

#### 3.3.9. Adherence to Monitoring Regimen

Table 3 shows the usage of the patients. Ninety per cent of the participants completed the full trial period and adhered to the monitoring regimen, while 10% of the participants did not complete the full trial regimen, as their data were not present on some days. Out of the 560 measurements that should have been submitted during the full trial period, eight (1.4%) were missing.

##### Missing Usage

There was missing usage on Days 5 and 8 by one couple who had to travel on those days and came back very late. One patient failed to take their measurement on Days 1 and 14 as he was busy with other commitments. On Day 8, one user failed to take their measurements due frustration as the batteries ran out before they could take their measurement and they had no spare batteries.

The highest number of events of missing usage on one day was five. During the focus group meetings, participants were asked about the reasons for non-usage. Participants reported:

“We were away for a few days and didn’t want to carry the equipment due to its bulkiness.” (FGD1).

“It wasn’t enjoyable; the cuff was too tight and sometimes painful.” (FGD2).

“We had other commitments.” (FGD3).

“The BP kept flashing indicating it needed replacement of batteries when switched on, then eventually it would switch itself off in the process due to battery drainage and we did not have replacement batteries.” (FGD1).

### 3.4. Phase 3: Post Trial Focus Groups

#### 3.4.1. Theme 1: Technology

##### Sub-Theme: Technology Design Preferences

User design preferences play a vital role towards technology acceptance. The needs for the elderly can be complex. When they were asked about what can be done to improve the technology, they mentioned improving battery lifespan, as they quickly drained. They suggested improvements of the BP meters with the ability to charge the batteries.

“Rechargeable blood pressure meter as the battery drains very fast, we had to change the batteries twice” (FGD1).

The number of devices was a major issue for many patients as they felt the whole system was bulky and suggested some improvements to the system.

“All in one compact case for storing the devices” (FGD1).

“Reduce the number of equipment’s” (FGD2).

Some patients expressed that proper hygiene is important for medical devices since the devices were exchanged between patients over the period. Ability to clean the devices was an important factor.

“Since they are medical devices which are exchanged between patients they should be easier to clean” (FGD3).

Some patients felt the cuff was too tight and expressed concerns about it and recommended that an easier to use cuff would be appropriate.

“Easier to use cuff” (FGD2).

Some patients who opted to take key fobs expressed concerns about the sensitivity of the key fobs suggested that the sensitivity of the reader should be improved to better read the key fobs.

“Improve sensitivity of the reader or the fob” (FGD3).

#### 3.4.2. Theme 2: Patient Characteristics

##### Sub-Theme: Trialling

In this study, it involves users having a lived experience with the technology and form judgement on ease of use. The participants were given the modified blood pressure monitor to use for a period of two weeks.

Majority of the participants expressed some level of satisfaction with usability of the system.

“Reasonably good” (FGD1).

“Overall good but there are some few areas which can be improved” (FGD2).

“No real issue with the NFC cards” (FGD1).

When the patients were asked what would influence them to use the technology, majority of the patients pointed to the ability of the technology to help them in managing their conditions and improving their lives.

“Incentive to improve my quality of life would really motivate in using this technology” (FGD1).

“Help in managing my condition better” (FGD2).

##### Sub-Theme 2: Attitude and Support

Majority of the patients chose to focus on the ability of the technology give them the opportunity to monitor their health.

“I believe this technology has its place, am willing to use it” (FGD3).

Some participants expressed that they relied on the support of their partners during the trial period, and their motivation was that they had their partners to motivate them and the exercise was for a good cause. “We motivated each other while using the kit and we would take our measurements together” (FGD1).

Some participants expressed how the support and influence of their partners was important during the trial period of the study.

“We would remind each other to take our measurements” (FGD3).

## 4. Discussions

### 4.1. Phase 1

The analysis of the data collected in Phase 1 of the study revealed findings that were categorised into three major themes concerning patients’ characteristics, technology and trust in clinicians. These findings are discussed in the following subsections.

#### 4.1.1. Patient Characteristics

The findings of the focus group discussions data revealed that there is a need to address issues associated with physiological characteristics of patients as a contributing factor to technology acceptance. Patients with physiological characteristics, such as memory problems, expressed concerns about their ability to remember to record properly their readings or perform certain tasks that involved them having to remember information. As people grow older, their ability to remember to perform certain tasks becomes a concern [20]. During the focus group discussions, the elderly patients expressed their concerns about using passwords or PINs as an identification technique. However, the current theoretical models on technology acceptance, such as TAM [21], do not address the issue of physiological characteristics as a determinant of technology acceptance among the elderly. These findings support the literature showing there is a lack of research on the investigation of the ways in which physiological characteristics affect technology usage and acceptance among the elderly [22]. Attitude affects a person’s behaviour by sifting through information and shaping the person’s perception of the world [23]. The literature shows that attitude affects a person’s behaviour through the process of sifting through information and shaping the person’s perception of the world [23]. In the case of elderly patients, attitude plays a significant role in technology acceptance. This was evident from the findings of the present study that showed that the majority of the patients expressed the desire to manage their conditions better as a major determinant in wanting to use the modified RPM device. It was also found that, after being diagnosed with the disease, the perception and attitude of patients towards things become changed and patients want to manage their conditions or even get better. These were the key influencers in them wanting to use the technology proposed in the present study. These findings contradict the argument of Venketesh [24] that attitude plays only a limited role in a person’s behavioural intention to use technology. In contrast, the present study showed that the appropriate attitude plays a significant role in a patients’ intention to use technology and it can be seen how patient attitudes were inclined towards the proposed technology given their appreciation of the benefit that might be given from its use. Patients were therefore open to the potential that it could provide by showing willingness to learn to use the proposed technology. The literature describes self-efficacy as a person’s belief that they have the capability to carry out a given action [25]. The present study noted that the elderly want to be in control of every aspect of their lives and they expressed the desire to be in control and have the ability to use technology without feeling less intelligent when using it. These findings are in agreement with earlier studies [25,26]. Ease of use is a known significant factor in technology acceptance among the elderly [25,27,28]. The findings of the present study showed that most of the patients did not experience issues with usability of the modified RPM device. However, it was found that some participants were concerned about making errors by mixing up their tags while they used the technology in their home environment. Patients using the wrong tag could potentially be a major issue, as measurements would end up in the incorrect patient record. These findings from Phase 1 helped in adjusting the technology to help prevent this error. This issue was resolved by making the tags easily distinguishable, in this case making the male user tag with a male picture icon or a distinguishable colour and a female tag with a female picture icon. This study further suggests the use of different types of tags or use clearly of identifiable marking to distinguish between tags with different purposes. In an environment, such as the home, errors such as using the wrong tag can easily happen; therefore, clear marking and using picture icons representing different sexes can play a significant role in avoiding mixing up of tags that might lead to errors. The market research methodology adopted in this study allowed patients to be involved in the design process of the modified RPM device. The concept of several types of identification technologies was presented to the patients to allow comparison and elicit comments on any advantages of disadvantages of each technology [29]. This provided a user-centred approach to the design process. Patients had the opportunity to express their feelings, beliefs and perceptions about the proposed technology and influence the design of the technology that they would receive in the pilot. 

Patient involvement in the design process of technology can play a significant role in technology acceptance. The needs of elderly people can be complex and involving them in the design process is one way to determine the functional requirements of a user-centred device. In the present study, issues concerning use and improvements were identified, which included having an additional set of batteries for the BP monitor and clear marking of tags to distinguish between users. These findings suggest a need for close collaboration between technology developers, clinicians and patients. Patients want to be more involved in important aspects of a MDT that is used in monitoring their health. Involving patients serves two purposes, firstly, it ensures patients are in control and secondly, it helps to identify underlying issues at early stages of the MDT development. This approach can ensure that the MDT is centred towards the needs of patients and so more likely to be easily accepted by the patients. These findings further show the enthusiasm of patients to be involved in the focus group interviews that were held to identify issues and improve the current RPM devices.

#### 4.1.2. Patient Education

Education can be an important tool in influencing adoption of MDT by elderly patients. The findings of the present study showed that the majority of the patients considered education as a major factor of their coping mechanism to manage their condition and attitude change. In the present digital era, where information is readily available, elderly people are becoming informed [30]. The present study found that elderly people use publicly available resources to become informed on how to manage their condition and gain knowledge on the things that matter most to them, including their health. These findings show that knowledge gained from training and product reviews can be a significant factor in technology acceptance among the elderly. This is supported in the literature; Ref. [31] claimed that training can be used to improve the level of literacy and educate the elderly, whilst at the same time reduce the level of anxiety caused by the technology, improve technology efficacy, and increase interest. The findings suggest that training patients in the use of the RPM technology and its benefits can play a significant role in technology acceptance among the frail elderly. The findings also show a key influencer in technology acceptance and use can be product reviews online [32]. It is evident that patients use such key aspects in determining their choices when it comes to use and acceptance of technology [32]. The present study also revealed that patients not only rely on the opinion of their doctor but also by seeking information on the conditions that affect them. These findings are in line with the study by Andreassen, ref. [30] which found that elderly people are likely to use technology when they want to gain knowledge on their health. The literature shows that elderly people are apprehensive towards the use of technology [4,5,33]. However, the findings in the present study show that this commonly held view that the elderly are afraid to use technology is not necessarily always the case. The present study showed that the majority of patients would be willing to use and be trained to use the proposed technology if they see it could be beneficial to them and would help them in managing their condition.

#### 4.1.3. Technology

Technology provides an interface between the patient and the clinician; thus, it plays a significant role in bridging the gap between the patient and the clinician. However, any underlying issues in relation to technology need to be clearly highlighted and addressed for technology to be easily accepted by patients. The present study showed that perceived security and security issues played little significance in the decision of the elderly patients to use the modified RPM device. It was also found that elderly patients have little interest in the security of RPM systems and they did not see the importance of security in transmission of their physiological measurements. These findings of the present study contradict the earlier studies that reported that security was an important aspect in HIT for patients [25,34]. This is not to say that security is not important, and aspects of security are considered in the present study.

The findings of the present study further revealed that that majority of the patients see the benefits of RPM technology, such as sharing and transmission of data electronically, to be of greater importance than the security issues. However, the present study showed that some patients would only want people with the right authority to have access to their health information [35].

#### 4.1.4. Cost

Cost is a significant factor in the acceptance of MDT by patients [36]. The present study found that patients have a major concern regarding the cost of point of care devices such as BP monitors. In addition, the findings of the present study showed that patients have become aware of the current crisis facing the NHS and that one way for the NHS to become able to sustain itself is through technology as a means of cutting cost. The ability of the proposed technology to serve a multi-user environment is one way the NHS can use it to cut costs, and this idea resonated well with the patients. However, current theoretical models, such as TAM, do not address the issue of cost as a key factor in technology acceptance by the elderly. These findings show cost as a predominant factor in determining whether elderly patients will accept to use the modified RPM device. These findings are in line with the findings by Venkatesh et al. [37,38,39]: the cost of medical devices is an important factor in the acceptance of technology by patients.

#### 4.1.5. Performance and Reliability

The present study showed that performance and reliability can play a major role in acceptance of technology among the frail elderly. The findings show that the ability of the device to work properly and send readings that are reliable are of great significance for acceptance of technology by the elderly, and agrees with the findings of others [34]. The findings of the present study revealed that performance of the BP monitor in terms of battery life is a major concern for elderly patients. If this concern is not addressed, then patient confidence will be undermined and might hinder efforts of adoption or result in the medical device being abandoned after some time. These findings are supported by findings from Cimperman et al. [25,34] that patients are likely to have negative views if technology is not reliable and dependable. The present study also showed that for a MDT to be accepted by patients it should have the ability to be useful in managing the medical conditions of the patient and it should also help in cutting costs for the healthcare provider. Additionally, the present study revealed that the ability to send physiological data electronically and having interventions done in a timely manner was an attractive aspect to the patients. The findings suggest that patient identification in RPM is very important because it can prevent patients receiving incorrect health and care interventions. These findings have bearings not only for MDT developers but also for the healthcare providers and funders. Moreover, the present study confirmed that to be of value for monitoring elderly patients the proposed technology needs to cater for multi-user environments such as homes where there is more than one person suffering from the same chronic disease [40,41].

#### 4.1.6. Clinician Trust

A positive relationship between clinician and patient plays a significant role in technology acceptance among the patients [42,43]. The present study showed that one of the most important factors that would influence the patients to use the proposed medical device technology was the clinician. In addition, patients believe clinicians make informed choices when it comes to their health [8]. However, the findings of the present study revealed that there was some degree of mistrust from some patients towards the clinicians, which was based on their personal experience that sometimes the doctors are not so keen in showing their medical records when they are required or only showing what is required [35]. 

The present study also revealed that patients have become more aware and they are using external resources to determine better ways to manage their condition, and technology is one such way. It was also found that some patients believe that the clinician is quick to administer medication but does not consider alternatives that can help patients, such as using MDT for self-management. 

### 4.2. Study Phase 2: NFC Trial

Phase 2 of the present study involved patients who were couples suffering from the same chronic disease participating in a lived experience with the modified RPM device. The identified main themes of findings in this phase of the study are discussed in the following sections.

#### 4.2.1. Technology

The findings of the present study showed that cost is a major determinant in acceptance of technology for elderly patients. The findings further revealed that patients support and consider that it would be sensible to use a cost saving technology that helps their healthcare provider, such as the NHS in England, which is currently going through many financial challenges [44]. In the UK, healthcare is provided to all of its legal residents free of charge, however, recently, there have been financial cuts in the NHS, and it must determine ways to streamline its operations [44]. The findings of this study suggest that the NHS can benefit from the use of the modified RPM devices to cut costs in environments with multi-users suffering from the same chronic disease by sharing the same medical device for self-testing and monitoring the same medical condition. A simple BP will cost around £30, while a BP monitor with NFC will have an additional cost of around £20. Therefore, it is significant to note that a modified RPM will be more advantageous in a care environment where there are multiple people with care needs, such as a care home [9].

#### 4.2.2. Technology Concerns

Experiences of the patients in the trial identified technological concerns. The findings of the present study showed that the patients had a major concern about the performance of the battery in the BP monitor, because it would only last a few days before the need for replacement. This could be a hindrance to this technology being accepted by the elderly, as they would have a concern over the financial cost involved in having to replace the batteries frequently. The present study also identified that the fragility of the USB dongle was a major concern among the patients. The USB dongle was used to create the interface between the BP monitor and the PC. The patients were particularly concerned about the circuits being visible because a lot of care had to be taken when connecting the USB dongle to the PC. The findings also revealed that some participants had noted that their key fob was not working properly. It can be very frustrating to a user when the technology is not working properly and it could deter use and acceptance. Elderly patients become frustrated easily and if they find that the technology is difficult and unreliable to use they will stop using it [45]. These findings suggest that immediate support must be available to the patients when they experience any technical issues and this could be valuable in helping them when they feel frustrated and so avoid them from stopping use of the technology [7,45].

Additional issues of technology experienced in the trial by the participants included the number of devices that they had to connect in order to be able to use the RPM system. Even though the connections were simple and there were only four devices, it proved to be a challenge, as many patients saw the system as bulky. This issue could be a major challenge for patients with cognition impairments; hence, they might have problems with MDT [22] such as misplacing certain medical devices and connecting various devices together. 

#### 4.2.3. Technology Design Preferences

The present study reveals that patients could be the source of new ideas about the design of MDT and suggests areas that need to be improved to make the technology more efficient and acceptable [46]. In this study, the patients suggested that BP monitors could include rechargeable batteries to reduce the frustration faced in changing the batteries after a very short time. For the issue of too many connections, the patients suggested that an all in one carry case could be provided for the equipment, as no proper storage was provided. An all in one carry case could be helpful to ensure that the patients do not lose the devices, support travel, and simplify carrying to/from the health centre when installing and retrieving. As the proposed technology used in the trial in the present study is still in prototype phase, future research will include reduction of the number of devices by integrating devices together and having a different form factor.

The findings further showed that the reader should be improved to ensure that when the user places their tag on the reader there is a confirmation by producing a sound. Although the current NFC reader confirms reading the tag by flashing the colour green when a tag is placed on it, producing a sound would improve reporting errors of use.

#### 4.2.4. Patient Characteristics and Technology Trialling

The present study showed that the characteristics of patients as the end users are important in the trial phase of a MDT [46]. In the present study, it was noted that the majority of the trial participants were satisfied with the level of usability of the proposed MDT. The level of satisfaction of the patients might be attributed to the low number of prompts used in the technology. It is however important to ensure that MDTs and RPM systems made for elderly people are easy to use and understand [47]. It is argued that the lived experience of patients and end users who are trialling a proposed MDT needs to be for a representative time, such as the two weeks in the present study, to best identify underlying limitations and other issues within the technology [48], which would not otherwise be identified if the patients only used it on one occasion.

The most noteworthy finding in this study was the support given by partners during the technology trial period of the study. The findings revealed that patients relied on the support of their partner to remind them to take their BP measurements, use the equipment correctly, motivate them to want to manage their condition, and support them in using the equipment. Each participant in the study played a unique role by influencing their partner to use the modified RPM device, as well as sharing beliefs and attitudes, offering guidance and support, and being a co-user. These findings provide empirical evidence that technology users benefit from support of family, as reported by earlier studies [47].

#### 4.2.5. Clinicians Trust

Clinicians play a vital role in influencing acceptance of technology by patients [39], and the findings of this study showed clearly that the patients were influenced to use the modified RPM device by the clinicians. This study found that there is a sense of trust from patients in their clinicians concerning making informed choices when it comes to recommendations towards factors that affect their health.

However, the same sentiment was not shared by all patients, as some wanted the clinicians to take an interest in the technology and link their systems with the proposed technology for it to be successful.

##### Issues Encountered by Users

This study revealed that the elderly can encounter issues whilst using the RPM system including the following.

##### Same Tag Used Twice on the Same Day

This error occurs when the same tag is used more than once on the same day. There may be several causes. Some patients experienced difficulties with the reader not recognising the key fob tag, which resulted in the patients using their partner’s tag when taking their measurement. Although it was stressed at the start of the study that patients should only use their own tag, patients believed it was more important to take the measurement, and so would use any tag. Alternatively, patients might have forgotten that they were supposed to use only their own tag. These findings suggest that future research should include having mechanisms in place to detect when the same tag is used more than once on the same day and issue an error message to the patient prompting them to use a different tag or wait for some time before taking another set of measurements. These findings are supported in other studies [22,45], which show that, as people grow older, their working memory decreases and this can make them forget. In addition, system complexity can easily cause frustration in use for elderly people. The findings also show that during a trial on MDT, situations can arise when a patient may use their partners’ card unintentionally. In this instance, the patient environment can be a contributory factor to this kind of error. For example, events or situations may lead to patients being confused or not in the right state of mind when taking measurements. For example, one couple had young grandchildren who visited them on Sundays, and they found it demanding to cope with playing with the children and telling them what they should do. The data showed how one partner of this couple had used the same tag to identify themselves on one Sunday. The BP measurements, taken one minute apart, clearly have distinct values, leading to the conclusion that the each had taken their measurement, but one had used the incorrect tag. These findings suggest that patients using the same equipment may be likely to experience this kind of error. Therefore, future research should include a mechanism to detect if the same card is used more than once within a short period, and impose a minimum time between measurements using the same card, unless the patient is instructed to take measurements on a more frequent basis.

##### Tag Not Placed Properly on the Reader

The findings show that this type of error occurs when patients failed to place their card properly on the reader. This type of error is highly likely to occur when the patient is suffering from physiological characteristics associated with the ageing process, such as shaking and tremors. This study observed that the male partner of one of the couples had this condition. The condition was noted at the time that the system was being set up in the patient’s home and that the patient had tremors and was shaking a lot. During analysis, it was found that the BP measurements of the male patient were being taken without identification, whereas the measurements of the wife were taken with her identification by the tag. It is assumed that this type of error could occur in this study either if the patient failed to place their card properly on the reader or failed to use the card. In the present study, this issue occurred on three occasions. These findings suggest that patients with similar physiological characteristics are highly likely to make the same error [49]. It is therefore important that there is a proper error reporting mechanism that can notify a patient when They have placed the tag correctly, have used the correct tag, and when a tag has not been read but a measurement is being made. This will allow the user to know when they have made an error. In the present study, the NFC reader had limited reporting capabilities, and was achieved through flashing coloured lights, which is not found sufficient by all people. These findings suggest that the needs of patients differ, especially frail elderly patients, and are different from younger and relatively less old patients; hence, their needs and requirements need special consideration in the development process of RPM.

##### Adherence to Technology Trial

The present study found that adherence to the protocol during the trial study by patients was generally good. However, some of the patients did not complete the full set of measurements. This was attributed to several factors, mostly that other commitments prevented them from taking some measurements. One couple reported that they found the equipment to be bulky and they could not carry it with them if they were away from home. Another reason related to the technical issue of battery life, i.e., batteries becoming exhausted would result in patients not following the daily monitoring regimen protocol. The study found that several issues can contribute to confusion and frustration in the users and affect adherence to the monitoring. Moreover, the findings show that, despite the significant interest of the participants in the trial of medical device technology, problems can arise due to personal and health related circumstances. Therefore, future research studies should determine how to ensure such issues do not arise.

The present study has identified significant factors that can affect the adoption of MDT for self-testing and RPM by elderly patients. These factors have not previously been incorporated in technology acceptance models, and therefore a new model of technology acceptance by elderly patients is proposed.

### 4.3. Proposing Senior Patients Technology Acceptance Model 

**Figure 2:**
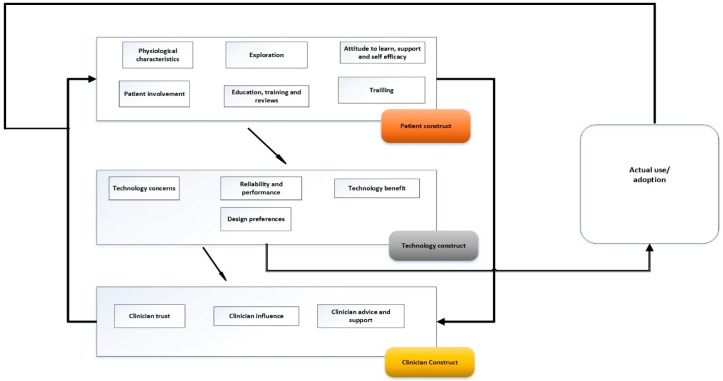
Senior Patients Technology Acceptance Model (SPTAM).

This study proposes a new framework to describe acceptance and adoption of technology by elderly patients: the Senior Patients Technology Acceptance Model (SPTAM) (Figure 2). SPTAM has been applied to the context of an elderly patient using medical device technology to take physiological measurements, such as BP readings, which are sent electronically to the clinician or healthcare provider, who in turn makes decisions on management, such as when to make an intervention. This model has been developed based on the themes identified in the data analysis of the focus group meetings and from the observations of elderly patients using the medical device technology developed for RPM. 

SPTAM captures the context of the elderly RPM user in an improved way, as it relates the technology acceptance factors to the adoption of the proposed technology. SPTAM identifies that there are several factors that influence technology acceptance by the elderly patients and that these factors are related to three themes: the elderly patient, the technology and the clinician. These three themes identified in the data collected in Phase 1 and Phase 3 of the study are incorporated as three constructs that have direct effect on each other in SPTAM. 

In SPTAM, the patient construct consists of the six sub-themes that were derived from the analysis of the data collected from the focus group interviews in Phase 1 and Phase 3 of the present study. This construct represents the patients’ beliefs, perceptions and needs, which influence their perception of technology and thus its acceptance; this will result in either acceptance or rejection of the technology. 

The patient construct consists of six factors identified from Phase 1 and Phase 3 (sub-themes): disease and physiological characteristics of the patient; patient’s attitude to learn and self-efficacy; trialling of technology; exploration; patient involvement in the MDT development process; and patient’s education, training and access to reviews/experiences of other users.

The technology construct has been placed at the centre of SPTAM because it creates an interface between the patient and clinician. The patients use technology to interact with the clinician through the mediation of sending their physiological measurements. In SPTAM, the technology construct has been used as the link between the patient and the clinician and embodies the concerns of the patient towards the technology, and thus influences their decision to adopt. 

The technology construct consists of five factors (sub-themes): concerns and issues, cost, reliability and performance, benefits, and design. The technology construct then relates these factors with the patient’s needs, beliefs, and perceptions, which are significant in technology acceptance.

Placing the technology construct at the centre of SPTAM emphasises the importance of patient engagement in the MDT design process and ensures that the technology proposed to the patient is user-centred and focused towards the needs of the patient. This aspect is expressed in the technology design preference factor, through which patients can recommend the changes they would like to be made in order for the technology to be more suitable for them, which has direct influence on acceptance. SPTAM also indicates how understanding the attitude of elderly patients towards the technology and the support that they might need in using the technology if they have physiological issues are very important considerations. At the same time, patient education, training, and access to other users’ reviews are very useful ways and means of improving the patients’ knowledge about the MDT. Providing the skills required needed to use the modified RPM device is also essential to eventual acceptance. For these reasons, the patient construct interacts directly with the technology construct in SPTAM.

The clinicians construct is the final construct included in SPTAM. This represents the influence that the clinicians have on the acceptance of technology through their interaction with the patients and their own attitude towards technology. The clinicians construct consists of three factors: patients’ trust in clinicians, clinicians’ influence, and clinicians’ advice and support. 

The clinician’s approach, through either active support or advice, will directly affect the patients’ attitude towards the use and acceptance of the MDT. This means that the patients are highly likely to accept use of the technology if they are influenced positively and effectively by the clinicians. 

Thus, SPTAM shows that the patient, the technology, and the clinician are the key determinants of the acceptance and adoption of MDT for home monitoring by patients.

#### 4.3.1. Advantages of SPTAM

SPTAM overcomes the limitations of previous technology acceptance models such as TAM and TRA by important additions:

- Including social norms within the patient construct;
- Recognising the importance of user involvement in the design and decision; and
- Recognising the interaction between key players, in this case the effect of the attitude of the clinician towards the technology and their approach to the patient.

#### 4.3.2. Study Limitations

The study has a number of limitations:

- The limited set of patients from only two similar geographic areas.
- The study was carried out in North London and Berkshire, generally considered as an affluent and rural area.
- Participants were mainly white and results might differ from people of other ethnic origin, culture and customs.
- The use of prototype technology resulted in comments that might not apply to the final form of the technology (e.g., after integration).

#### 4.3.3. Study Strengths

The study has a number of strengths:

- Although the literature of Nielsen [50] specifies five participants may be sufficient to identify 80% to 85% of problems, in this study, problems were identified during Phase 1 and further problems were identified during Phase 3. This would justify the use of the much larger group of 40 participants (20 couples) to test the RPM technology system through a lived experience.
- The extended period of testing that matched the real-world use of the technology provided a robust testing of NFC for identification of RPM devices and helped to identify many usability issues.

The number of people with chronic disease is increasing in many countries, including the UK, which is placing pressure on health and social care services. Due to limited resources and capacity, health services could fail to meet the health and care needs of a growing number of people with chronic disease; thus, there is an urgent need to address the current crisis in health and social care. 

Telehealth has been seen as a solution for managing the emerging health and care crisis due to the burden of chronic disease. However, despite developments and improvements in telehealth over the years, the current RPM devices still have limitations, one of which is the limit on the number of people who can use the same RPM device. In addition, there is a threat of people willing to take advantage of security issues, which has become an issue in healthcare. Thus, the lack of familiarity of security and balancing between providing care and security is becoming a challenge.

In addition, elderly patients with chronic disease can gain significant benefit from the use of telehealth. It is therefore important to identify any underlying issues that might hinder elderly patients from accepting and using a telehealth system. However, limitations remain and, if not addressed, may be a barrier to successful implementation of telehealth. Therefore, it is imperative to identify and address the limitations in order to improve telehealth systems such as RPM. Literature already identifies limitations of current RPM devices, including aspects such as a limit to the number of people who can use a single device [8].

This study investigated the current limitations in telehealth. It has identified and focused on security issues and ways to improve identification techniques for patients using RPM devices.

The study objectives were:

- Identify and compare identification techniques for a multi-user environment where two or more people suffering from the same chronic disease are sharing a single device.
- Select and test an identification technique for a multi-user environment.
- Identify elderly patients’ beliefs, needs, and perceptions about the use of NFC for identification in RPM devices.
- Identify elderly patients’ behavioural usage and issues associated with using NFC for identification in RPM devices through a lived experience.
- Make recommendations for improved design of RPM devices.

In the technology trial phase, all patients were given a modified RPM device with NFC to use for a period of two weeks to observe their behavioural usage of the device.

This study has identified a problem list (from Phase 2 and Phase 3), design preferences, and errors in the trial study that could be used to improve the RPM system before it would be implemented in final form.

For those patients that adhered to the full monitoring regimen protocol, the findings show that it was their desire to be involved in something that would be beneficial, and help them to be in control and manage their own health and medical condition better. The findings revealed that the ability of patients to manage their condition and improve their lifestyle with the proposed technology was a major influence, as the patients saw the benefits of what the proposed technology could achieve.

The findings of the study contradict work of others [39] that suggests elderly adults are not ready to adopt HIT. However, it is important to explain the benefit of the technology to the patients for the technology to be easily acceptable to them.

One of the strengths of this study was that user identification using NFC in RPM devices was tested through the lived experience of the patients taking the devices and using them in their home environments, which generated a sense of how the devices would behave in their normal environment. This provided detailed and accurate information on the personal experiences of the patients and the errors and problems that occurred, which would not have been achieved if the technology trial had been conducted only in a controlled setting or for a short period.

The present study provides empirical evidence that ease of use, cost, and technology benefit are significant determinants of technology acceptance among the elderly. Therefore, it is significant that the benefit of the technology is made clear to the patient in order to increase the likelihood of acceptance of the technology.

Education, training and reviews can also enhance the knowledge of the patient about the benefits of using the technology.

The findings in this study show that involving patients in the early stages of technology development can yield positive results. This study gave the patients a voice by allowing them to participate in the design process of the MDT. The patients were given the opportunity to articulate their concerns and perceptions in advance of the trial and then to specify their design preferences after the lived experience with the proposed technology. Thus, resulting in a user-centred approach which can increase the likelihood of adoption. Medical device manufacturers can use the results of this study to understand the elderly patients better to ensure technology presented to them is easily acceptable.

## 5. Conclusions

The present study confirmed that NFC is an effective method to identify multiple people sharing a single RPM device. It was found that involving patients in the early stages of medical device technology development and evaluation can yield positive results including identification of patients’ (end users) concerns, beliefs, preferences, and issues at early stage, which can help to develop more user-centred technology. This study achieved this by placing the patient at the centre of an iterative design process by investigating underlying issues, concerns and preferences of the patients at the outset of the process, seeking feedback during the process, and through evaluation at the end. This approach will help ensure that any proposed technology is usable and acceptable to the patients. 

The findings in this study show that NFC was the preferred identification technique by the majority of the participants compared to other techniques such as PINS and fingerprint. Participants were apprehensive about fingerprints as they saw this as a method used by the government for criminal profiling. For PINS, participants were concerned about forgetting them. The majority of the patients were familiar with NFC, which made it easier for them to relate to it, making it an ideal choice. However, despite NFC showing a lot of potential for being the most ideal identification technique, participants raised concerns about mixing their tags up with their partner’s. Participants were also apprehensive about the use of NFC wristband as it reminded some of them being hospitalised. There were some practical issues such as use by patients with tremors and shaky hands.

This study found elderly patients were eager to use the RPM as they believed it could help them manage their conditions and provide the possibility to improve. 

Nevertheless, there needs to be a close collaboration between medical device manufacturers and patients to determine the concerns and issues from the patients’ perspective. By closely collaborating with patients, MDT manufactures can understand the patients’ beliefs, behaviours, needs, and limitations, which can help develop better MDT.

## Figures and Tables

**Table 1:** Demographics.

Age (Years)	Gender	Participants
65–75	M	8
	F	10
75–85	M	8
	F	7
86–95	M	4
	F	3
Total		40

**Table 2:** The usage errors during the trial period.

Errors Made (%)	Day													
	1	2	3	4	5	6	7	8	9	10	11	12	13	14
Multiple Use of the Same Card	2.5	0	0	0	5	0	0	7.5	0	0	0	0	0	2.5
Tag not Placed Properly on the Reader	2.5	5	2.5	0	5	2.5	0	2.5	0	0	2.5	0	2.5	0
Total	5	5	2.5	0	10	2.5	0	10	0	0	2.5	0	2.5	2.5

**Table 3:** Usage by the patients.

Missing Usage (%)	Day													
	1	2	3	4	5	6	7	8	9	10	11	12	13	14
	2.5	0	0	0	5	0	0	7.5	2.5	0	0	0	0	2.5
